# Etiology of Diarrhea Requiring Hospitalization in Bangladesh by Quantitative Polymerase Chain Reaction, 2014–2018

**DOI:** 10.1093/cid/ciaa840

**Published:** 2020-06-27

**Authors:** Mami Taniuchi, Kamrul Islam, Md Abu Sayeed, James A Platts-Mills, Md Taufiqul Islam, Md Imam Ul Khabir, Muntasir Rahman, Zahid Hasan Khan, Yasmin Ara Begum, Farhana Khanam, Ashraful Islam Khan, Jie Liu, Eric R Houpt, Firdausi Qadri

**Affiliations:** 1 Division of Infectious Diseases and International Health, University of Virginia, Charlottesville, Virginia, USA; 2 Mucosal Immunology and Vaccinology Unit, Infectious Diseases Division, International Centre for Diarrhoeal Disease Research, Bangladesh (icddr,b), Mohakhali Dhaka, Bangladesh

**Keywords:** diarrheal diseases, Bangladesh, surveillance, TAC

## Abstract

**Background:**

Diarrhea remains a major public health problem and characterization of its etiology is needed to prioritize interventions. However, most data are from single-site studies of children. We tested samples from participants of any age from 11 geographically diverse hospitals in Bangladesh to describe pathogen-specific burdens of diarrhea.

**Methods:**

We utilized 2 existing diarrhea surveillance systems: a Nationwide network at 10 sentinel hospitals and at the icddr,b hospital. We tested stools from enrolled participants and nondiarrheal controls for enteropathogens using quantitative polymerase chain reaction and calculated pathogen-specific attributable fractions (AFs) of diarrhea.

**Results:**

We analyzed 5516 patients with diarrhea and 735 controls. Overall, rotavirus had the highest attributable burden of diarrhea (Nationwide AF, 17.7%; 95% confidence interval [CI], 14.3–20.9%; icddr,b AF, 39.9%; 38.0–41.8%), followed by adenovirus 40/41 (Nationwide AF, 17.9%; 95% CI: 13.9–21.9%; icddr,b AF, 16.6%; 95% CI, 14.4–19.4%) and *Vibrio cholerae* (Nationwide AF, 10.2%; 95% CI, 9.1–11.3%; icddr,b AF, 13.3%; 95% CI: 11.9–15.1%). Rotavirus was the leading pathogen in children <5 years and was consistent across the sites (coefficient of variation = 56.3%). Adenovirus 40/41 was the second leading pathogen in both children and adults. *Vibrio cholerae* was the leading pathogen in individuals >5 years old, but was more geographically variable (coefficient of variation = 71.5%). Other attributable pathogens included astrovirus, norovirus, *Shigella*, *Salmonella*, ETEC, sapovirus, and typical EPEC.

**Conclusions:**

Rotavirus, adenovirus 40/41, and *V. cholerae* were the leading etiologies of infectious diarrhea requiring hospitalization in Bangladesh. Other pathogens were important in certain age groups or sites.

## Teaser

Patients of all ages were enrolled in the sentinel surveillance from 11 Bangladesh hospitals. Rotavirus, adenovirus 40/41, and *Vibrio cholerae* were the leading causes of diarrhea across age and geography with variations in frequency. Other pathogens were important in certain ages or sites.

**(See the Editorial Commentary by Alkan and Alkan on pages e2500–1.)**

Diarrhea is estimated to be the eighth leading cause of mortality globally [1]. Defining pathogen-specific burdens of disease is critical for prioritizing the development of pathogen-specific interventions, including vaccines. Most etiology studies have focused on children, in whom disease burden is highest [2]; however, a substantial disease burden also exists in adults [1]. Additionally, single surveillance sites are often considered representative of entire countries, even though this involves extrapolation across diverse populations [3].

Estimates of etiology-specific diarrhea in children under 5 years have recently improved with the application of molecular diagnostics, which offer increased sensitivity and, via quantification, resolution to attribute etiology to pathogens detected in diarrheal stools [4, 5]. However, these diagnostics and analytic approaches have not been applied to studies of adults. Finally, even in children under 5 years, there have been limited studies using molecular methods to investigate diarrhea requiring hospitalization, the subset of diarrhea considered to the best proxy for fatal diarrhea [6, 7].

In this study, we examined the etiology of diarrhea requiring hospitalization in patients of all ages in 2 surveillance systems, including 11 broadly representative hospitals in Bangladesh. We utilized quantitative polymerase chain reaction (PCR) to detect enteric pathogens directly in stool with high sensitivity [8]. To adjust for high rates of asymptomatic carriage of enteropathogens in high-transmission countries such as Bangladesh, we also tested stool samples from control patients across all ages without diarrhea. We then calculated the attributable fraction (AF) of hospitalized diarrhea by pathogen and across ages and populations.

## METHODS

### Study Design

**Figure 1.**
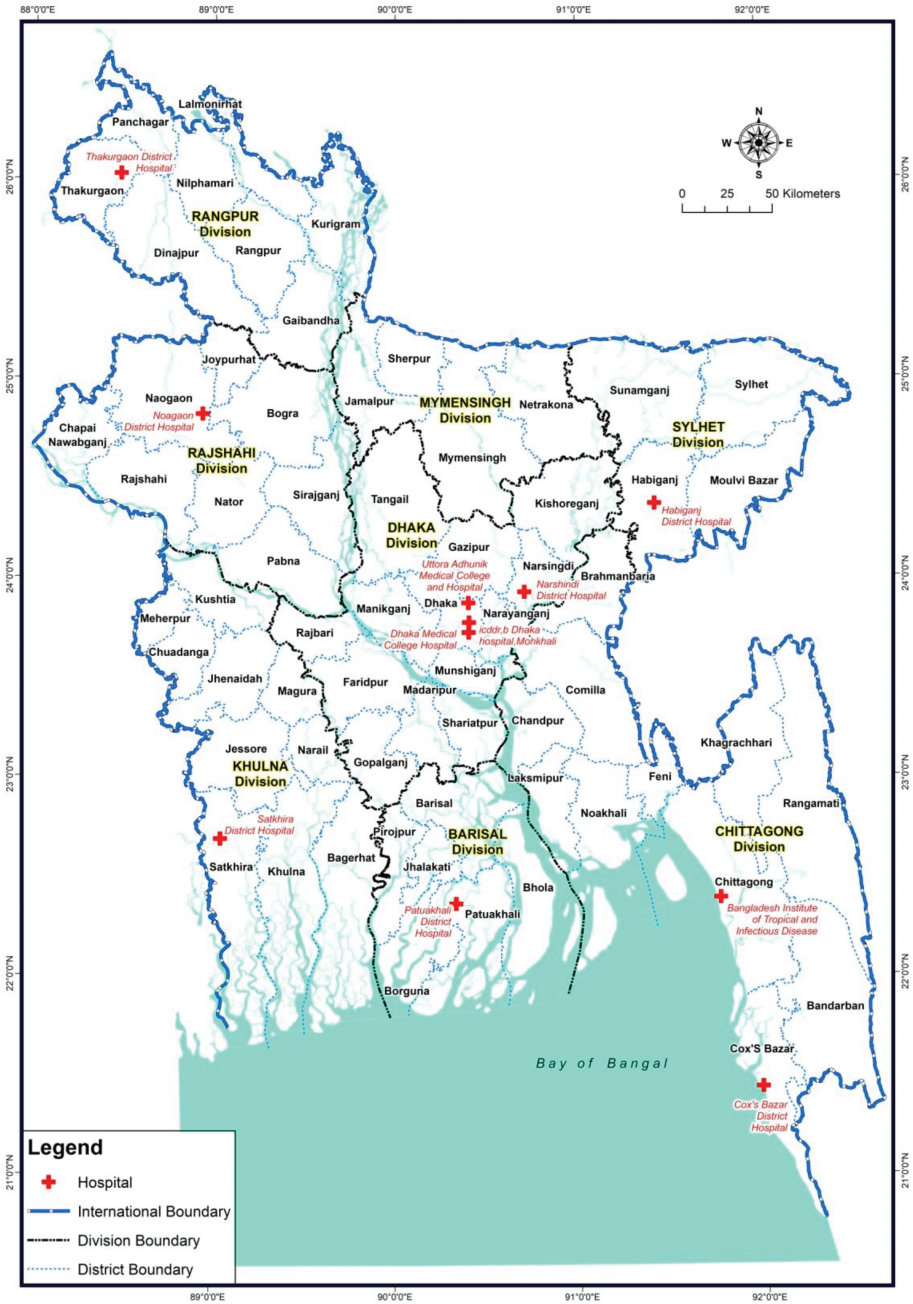
Map of the sentinel surveillance sites across Bangladesh. Study participants were enrolled from 11 sentinel sites indicated by red crosses: the Nationwide surveillance included 10 hospitals and the 2% surveillance was carried out at the icddr,b Dhaka hospital. Abbreviation: icddr,b, International Centre for Diarrhoeal Disease Research, Bangladesh.

This study was conducted using 2 established diarrhea surveillance systems in Bangladesh: (1) the Nationwide surveillance network, conducted at 10 sentinel sites in collaboration with the Institute of Epidemiology Disease Control and Research [9], where the first participant under 5 years of age and first participant over 5 years of age were enrolled each week at each hospital, and (2) the ongoing 2% diarrheal surveillance at International Centre for Diarrhoeal Disease Research, Bangladesh (icddr,b), hospital in Dhaka [10], in which a sample is collected from every 50th patient admitted with diarrhea. All the sentinel sites are shown on the map in Figure 1. Both systems included patients of any age presenting with diarrhea. In children aged less than 2 months, diarrhea was defined as a change in stool habits from a usual pattern in terms of frequency (more than the usual number of purging) or nature of stool (more water than fecal matter). In all other age groups, diarrhea was defined as 3 or more loose or liquid stools within 24 hours or 3 or fewer loose/liquid stools causing dehydration in the last 24 hours.

Patients who met the case definition and had no other severe comorbidity (eg, severe acute respiratory illness, acute cardiovascular symptoms, or severe acute neurological disorder) were enrolled by the physician. From all participants, demographic and clinical information such as age, gender, duration of diarrhea, dehydration status, vomiting, and abdominal pain was collected. Dehydration status was defined as none, some, and severe following the World Health Organization criteria [11]. A stool specimen was obtained from all participants.

### Selection of Control Specimens and Collection

Control participants were selected from patients presenting to care and without enteric symptoms (ie, nausea, vomiting, abdominal pain, or diarrhea) in the prior 2 weeks. For the Nationwide surveillance, the first gender-matched individual presenting to the site within 1 year of age was chosen as the control participant for every fifth case under 5 years old, and the first gender-matched individual presenting to the site within 5 years of age was chosen as the control participant for every fifth case over 5 years old. For the icddr,b surveillance, for every other case an age- and gender-matched control participant was identified from a nearby outpatient clinic (Mirpur area of Dhaka, Bangladesh). Age matching was done to the nearest year for children younger than 5 years of age and to the nearest 5 years for all other cases.

### Microbiological Studies

The use of TaqMan Array Cards (TAC; Thermo Fisher, Carlsbad, CA) to perform quantitative PCR testing for a broad range of diarrheal pathogens has been previously described [12]. Briefly, bacteriophage MS2 for RNA targets and phocine herpesvirus for DNA targets were added during total nucleic acid extraction to monitor nucleic acid extraction and amplification. Extraction blanks were included to monitor for laboratory contamination. All detections with a cycle threshold greater than or equal to 35 were considered negative. Valid results required proper control results for negative results, exclusion of contamination for positive detections, and the absence of quality-control flags from the PCR run analysis software. The pathogen targets tested by TAC for this study are indicated in Supplementary Table 1. Valid samples were defined as samples with valid results for all pathogens included in the etiology analysis.

### Data Analysis

Pearson’s chi-square test was used to compare dichotomous characteristics between groups. To estimate pathogen-specific burdens of diarrhea, we used the population AF, which assigns etiology based on the quantity-specific strength of association between pathogen detection and being a diarrheal case. Specifically, for each pathogen, we fit a logistic regression model to the entire dataset with valid TAC results for all included pathogens, with an outcome of case/control status and predictors of sex, age category, surveillance network, and the quantity of each pathogen, as well as interactions between the pathogen of interest, and both age category and surveillance network. We next calculated AFs by summing the pathogen-specific AF for each episode (AFe) across each of the *j* cases, namely ∑_1__j_(1/*j* *AF_i_)y, where *AFe*_i_ = 1 – 1/*OR*_i_, and *OR*_*i*_ is the quantity-, age- and network-specific odds ratio derived from the regression model. To estimate the variance for the model-based attribution, the odds ratios were estimated 1000 times using random perturbations of the model coefficients in accordance with their sampling variance-covariance. The point estimate of the AF was calculated using the original model coefficients, and 95% confidence intervals were derived from the 2.5th and 97.5th quantiles of the AF distribution. The same approach was used to calculate AFs for subgroups of cases by age, network, dehydration status, and hospital. Clinical characteristics were compared between subgroups using the Wilcoxon rank-sum test with continuity correction. To assess the consistency of pathogen between sites in the Nationwide network, we calculated a coefficient of variation for each pathogen as the standard deviation of the AF point estimates for the Nationwide sites divided by the mean of the AF point estimates. All analyses were performed using R 3.5.1 (R Foundation for Statistical Computing, Vienna, Austria [2018]).

## RESULTS

In the 2% surveillance of the icddr,b hospital, 3154 patients were enrolled between 5 August 2014 and 27 June 2017, of whom 3015 (95.6%) had samples available for testing and 3000 (99.5%) had valid results. In the Nationwide surveillance network, 2532 patients with diarrhea enrolled between 6 February 2014 and 29 June 2018 had samples available for testing and 2516 (99.4%) had valid results. A broad age range was represented, with approximately half of all cases in children less than 5 years of age (Table 1, Supplementary Figure 1). Severe dehydration was more common in the icddr,b hospital surveillance (27.9% of cases vs 5.8% in the Nationwide network, *P* < .001), and these cases had a shorter duration of diarrhea prior to hospital admission (mean ± standard deviation: 1.8 ± 0.8 in the icddr,b surveillance vs 2.8 ± 1.5 in the Nationwide network; *P* < .001). Other clinical characteristics were similar between the networks. For the icddr,b surveillance, 493 controls were enrolled, of whom 491 (99.6%) had valid results, whereas for the Nationwide surveillance network, 221 controls were enrolled, of whom 220 (99.5%) had valid results. For the icddr,b hospital surveillance, cases were older than controls (*P* = .002), but had a similar sex distribution (*P* = .417), while for the Nationwide surveillance, cases were older than controls (*P* < .001) and more likely to be female (*P* = .009) (Table 1).

**Table 1.** Characteristics of Enrolled Cases and Controls with Valid Quantitative Polymerase Chain Reaction Testing Results

	icddr,b 2% Surveillance		Nationwide Surveillance	
	Cases (n = 3000)	Controls (n = 491)	Cases (n = 2516)	Controls (n = 220)
Demographics				
Date range	6 August 2014–27 June 2017	2 August 2016–5 July 2017	6 February 2014–29 June 2018	19 June 2016–11 July 2018
Age category				
<12 months	950 (31.7)	144 (29.3)	488 (17)	66 (30)
12–23 months	401 (13.4)	101 (20.5)	355 (12.3)	37 (16.8)
24–59 months	109 (3.6)	20 (4.1)	134 (4.7)	15 (6.8)
5–17 years	148 (4.9)	21 (4.3)	150 (5.2)	17 (7.7)
18–55 years	1241 (41.4)	178 (36.2)	1159 (40.3)	64 (29.1)
>55 years	151 (5)	27 (5.5)	230 (8)	21 (9.5)
Female sex	1248 (41.6)	198 (40.3)	1129 (44.9)	78 (35.5)
Clinical characteristics				
Duration, days	1.8 ± 0.8	N/A	2.8 ± 1.5	N/A
Dehydration^a^				
None	983 (32.8)	N/A	922 (32)	N/A
Some	1179 (39.3)	N/A	1426 (49.5)	N/A
Severe	838 (27.9)	N/A	168 (5.8)	N/A
Vomiting	1007 (33.6)	N/A	989 (39.3)	N/A
Abdominal pain	1762 (58.7)	N/A	1563 (62.1)	N/A

Data are presented as means ± standard deviations for continuous variables and n (%) for dichotomous variables.

Abbreviations: icddr,b, International Centre for Diarrhoeal Disease Research, Bangladesh; N/A, not available.

^a^Based on the World Health Organization dehydration scale.

**Figure 2.**
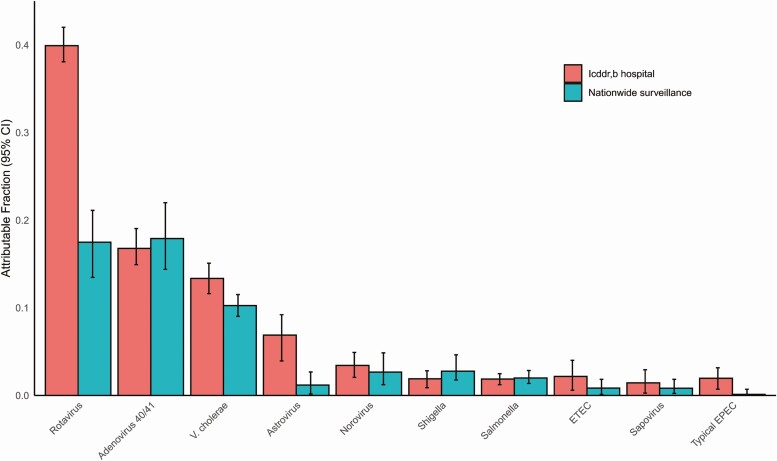
Pathogen-specific attributable fractions of diarrhea requiring hospitalization using quantitative molecular diagnostics in each surveillance network. Error bars denote 95% CIs. Abbreviations: CI, confidence interval; EPEC, enteropathogen *Escherichia coli*; ETEC, enterotoxigenic *Escherichia coli*; icddr,b, International Centre for Diarrhoeal Disease Research, Bangladesh.

**Figure 3.**
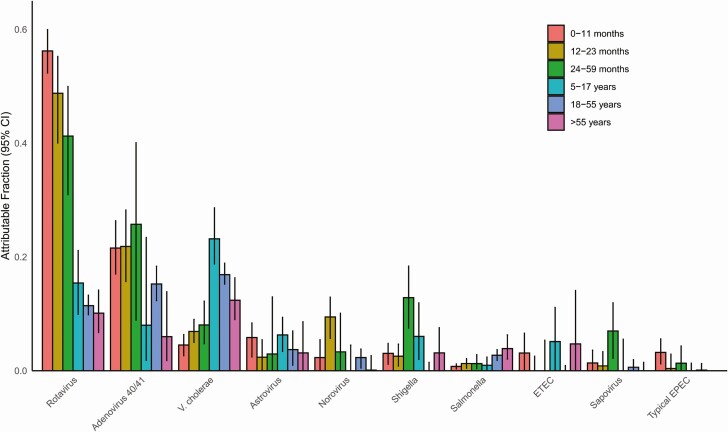
Pathogen-specific attributable fractions of diarrhea requiring hospitalization using quantitative molecular diagnostics by age category, both surveillance networks combined. Error bars denote 95% CIs. Abbreviations: CI, confidence interval; EPEC, enteropathogen *Escherichia coli*; ETEC, enterotoxigenic *Escherichia coli*.

**Figure 4.**
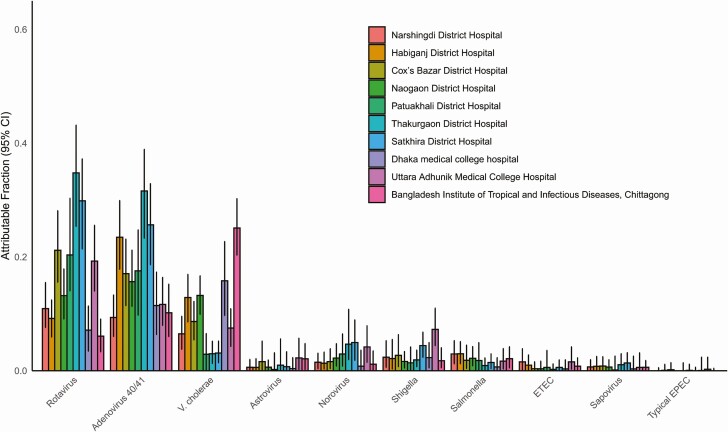
Pathogen-specific attributable fractions of diarrhea requiring hospitalization using quantitative molecular diagnostics in each of the 10 sites of the Nationwide surveillance system. Error bars denote 95% CIs. Abbreviations: CI, confidence interval; EPEC, enteropathogen *Escherichia coli*; ETEC, enterotoxigenic *Escherichia coli*.

We utilized the prevalence and quantity of pathogens in both cases and controls (Supplementary Figure 2) to develop attribution models for specific pathogens. Ten pathogens had an overall AF of at least 1%. The combined overall AFs for these 10 pathogens was 88.8% for the icddr,b surveillance and 56.2% for the Nationwide surveillance. Rotavirus, adenovirus 40/41, and *Vibrio cholerae* O1 were the leading etiologies of diarrhea in both networks (Figure 2). Indeed, these 3 organisms were attributed as the cause of 69.8% of the diarrhea in the icddr,b surveillance network and 45.8% in the Nationwide surveillance network. The burden of rotavirus and adenovirus 40/41 was particularly high in children under 2 years of age, while *Shigella* peaked in 2- to 5-year-olds (Figure 3). For enterotoxigenic *Escherichia coli* (ETEC), a peak was seen in children aged 5–17 years as well as those over 55 years. *Vibrio cholerae* was the leading pathogen in cases older than 5 years of age. There was some variation in the etiology between the 10 hospitals of the Nationwide network, particularly for cholera (coefficient of variation: 71.5%) relative to rotavirus (56.3%) or adenovirus 40/41 (42.7%) (Figure 4). *Vibrio cholerae*, rotavirus, and adenovirus 40/41 accounted for 82.4% of episodes presenting with severe dehydration (Supplementary Figure 3). After these top 3 pathogens, there was some variation in etiology between the 2 surveillance networks, including a higher incidence of astrovirus in the icddr,b surveillance. Other attributable pathogens included norovirus, *Shigella*, *Salmonella*, ETEC, sapovirus, and typical enteropathogen *E. coli* (EPEC). Significant seasonal variation in pathogen-associated diarrhea was seen for rotavirus, cholera, and adenovirus 40/41 (Supplementary Figure 4).

As a sensitivity analysis for the attribution modeling, we also estimated attribution using previously collected controls from the Global Enteric Multicenter Study (GEMS) and Etiology, Risk Factors, and Interactions of Enteric Infections and Malnutrition and the Consequences for Child Health and Development (MAL-ED) cohort studies for children under 5 years of age, and results were similar (Supplementary Figure 5).

## DISCUSSION

In this multiyear, multisite analysis of the etiology of diarrhea requiring hospitalization across diverse populations in Bangladesh, we consistently identified 3 pathogens as the leading etiologies: rotavirus, adenovirus 40/41, and *V. cholerae*. Vaccines are available for rotavirus and cholera. Cholera control is a recognized priority, and our results with molecular testing here are largely consistent with the established, culture-based surveillance [9]. The finding that adenovirus 40/41 was a major cause of diarrhea, including dehydrating diarrhea, across all ages and geographies has only recently been appreciated, primarily due to the application of quantitative molecular diagnostics [4, 5]. These data suggest that it is the second-leading cause of severe diarrhea in the youngest children at highest risk for poor outcomes, and further research is needed to characterize the molecular epidemiology, virology, and pathogenesis of this virus and identify pathogen-specific strategies for controlling the burden of disease.

Our study included 3 hospitals in Dhaka, one of the most densely populated urban areas in the world, and 8 other hospitals throughout Bangladesh. Rotavirus, adenovirus 40/41, and *V. cholerae* were the top 3 etiologies of diarrhea at 8 of 11 hospitals, while norovirus was the third-leading etiology of diarrhea for the remaining 3 hospitals. There was no clear rural–urban gradient in etiology. Other important pathogens included astrovirus, ETEC, and typical EPEC, particularly at icddrb, and *Shigella*, *Salmonella*, and sapovirus, which were seen across the networks and hospitals. This broadly suggests that relatively sparse surveillance is sufficient to define the leading etiologies of diarrhea in such settings.

Some pathogens that have been identified as leading causes of diarrhea in children under 5 years had negligible attribution as etiologies of diarrhea requiring hospitalization in this network. *Campylobacter jejuni* has been identified as a major etiology of community diarrhea, but appears to cause relatively mild disease, and thus is less common in the subset of diarrhea requiring hospitalization [5, 13]. *Cryptosporidium*, while clearly an important cause of severe, dehydrating diarrhea, had a comparatively low burden of diarrhea in Bangladesh sites in both the GEMS and the MAL-ED cohort study [4, 5].

Most studies of diarrhea etiology in low-resource settings, including Bangladesh, have focused on children under the age of 5 years. To our knowledge, this is the first study to apply attribution modeling using carefully identified nondiarrheal controls with quantitative molecular diagnostic testing to the etiology of diarrhea in adults in a low-resource setting. We found that *V. cholerae*, rotavirus, and adenovirus 40/41 in that order were the leading etiologies of diarrhea requiring hospitalization in adults. While *Shigella* and norovirus completed the top 5 etiologies in children, *Shigella* was not particularly attributable in adults. This runs contrary to recent data suggesting that *Shigella* is a major cause of fatal diarrhea in adults in these settings [13]. Importantly, the prevalence of *Shigella* in nondiarrheal stools continued to increase with age, reaching 12–33% in adulthood. Naturally, this reduced the attribution of *Shigella* as etiologic when detected in adults. Previous estimates have relied upon models derived from children under 5 to attribute etiology in adults [14]; however, this extrapolation is therefore uncertain. We believe that additional data are needed to model and understand the etiology of diarrhea in adults for highly endemic pathogens.

In conclusion, rotavirus, adenovirus 40/41, and *V. cholerae* were the most important diarrheagenic pathogens across age and geography in Bangladesh. Other pathogens were important in certain ages or regions. There is an urgent need to accelerate the roll-out of rotavirus and cholera vaccines in the national immunization program, which may significantly reduce the burden. Further research is needed on adenovirus diarrhea.

## Supplementary Data

Supplementary materials are available at *Clinical Infectious Diseases* online. Consisting of data provided by the authors to benefit the reader, the posted materials are not copyedited and are the sole responsibility of the authors, so questions or comments should be addressed to the corresponding author.

ciaa840_suppl_Supplementary_Material

## Notes

***Acknowledgments.*** The authors thank all the patients and their family who participated in the study. The icddr,b is grateful to the governments of Bangladesh, Canada, Sweden, and the United Kingdom for providing core/unrestricted support.

***Financial support.*** This work was supported by the Bill and Melinda Gates Foundation (grant number OPP1131107 to F. Q.).

***Potential conflicts of interest.*** The authors: No reported conflicts of interest. All authors have submitted the ICMJE Form for Disclosure of Potential Conflicts of Interest.
