# Supplementary material for: Etiology of Diarrhea Requiring Hospitalization in Bangladesh by Quantitative Polymerase Chain Reaction, 2014–2018
Source: Clin Infect Dis. 2020 Jun 27;73(9):e2493–9. doi: 10.1093/cid/ciaa840 (PMC8563176; doi:10.1093/cid/ciaa840)

Supplemental Table 1. qPCR assays included on TAC

|  | Pathogen | Reference |
| --- | --- | --- |
| Virus | Adenovirus F | (1) |
|  | Adenovirus | (2) |
|  | Astrovirus | (2) |
|  | Norovirus GI | (2) |
|  | Norovirus GII | (2) |
|  | Rotavirus | (2) |
|  | Rotavirus G-types | (3) |
|  | Rotavirus P-types | (3) |
|  | Sapovirus | (1) |
| Bacteria | EAEC | (2) |
|  | EPEC | (2) |
|  | ETEC | (2) |
|  | ETEC CFA/I | (4) |
|  | ETEC CS1/PCFO71 | (4) |
|  | ETEC CS2 | (4) |
|  | ETEC CS3 | (4) |
|  | ETEC CS5 | (4) |
|  | ETEC CS6 | (4) |
|  | STEC | (2) |
|  | *Aeromonas* | (1) |
|  | *Bacteroides fragilis* | (1) |
|  | *C. jejuni/C. coli* | (2) |
|  | *Campylobacter* spp. | (1) |
|  | *Clostridium difficile* | (1) |
|  | *Helicobacter pylori* | (1) |
|  | *Mycobacterium tuberculosis* | (1) |
|  | *Salmonella enterica* | (1) |
|  | *Shigella*/EIEC | (2) |
|  | *S. flexneri* | (5) |
|  |  | (5) |
|  |  | (5) |
|  | *S. sonnei* | (5) |
|  | *Vibrio cholerae* | (1) |
| Fungi | *Encephalitozoon intestinalis* | (1) |
|  | *Enterocytozoon bieneusi* | (1) |
| Protozoa | *Cryptosporidium* spp. | (2) |
|  | *Cryptosporidium hominis* | (1) |
|  | *Cryptosporidium parvum* | (1) |
|  | *Entamoeba histolytica* | (2) |
|  | *Cyclospora cayetanensis* | (1) |
|  | *Cystoisospora belli* | (1) |
| Helminth | *Ancylostoma duodenale* | (1) |
|  | *Ascaris lumbricoides* | (1) |
|  | *Necator americanus* | (1) |
|  | *Strongyloides stercoralis* | (1) |
|  | *Trichuris trichiurs* | (2) |

1. Liu J, Gratz J, Amour C, Nshama R, et al. Optimization of quantitative PCR methods for enteropathogen detection. PLoS ONE, **2016**, 11(6): e0158199

2. Liu J, Gratz J, Amour C, et al. A laboratory-developed TaqMan array card for simultaneous detection of 19 enteropathogens. J Clin Microbiol, **2013** 51(2): 472-480.

3. Liu J, Lurain K, Sobuz SU, et al. Molecular genotyping and quantitation assay for rotavirus surveillance. J Virol Methods, **2015** Mar; 213(1): 157-163

4. Liu J, Silapong S, Jeanwattanalert P, et al. Multiplex real time PCR panels to identify fourteen colonization factors of enterotoxigenic Escherichia coli (ETEC). PLoS ONE, **2017**, 12(5): e0176882

5. Liu J, Platts-Mills JA, Juma J, et al. Use of quantitative molecular diagnostic methods to identify causes of diarrhoea in children: a reanalysis of the GEMS case-control study. Lancet. **2016** Sep: 388:1291-301

Supplemental Figure 1. Age distribution of surveilled diarrhea cases by surveillance network.


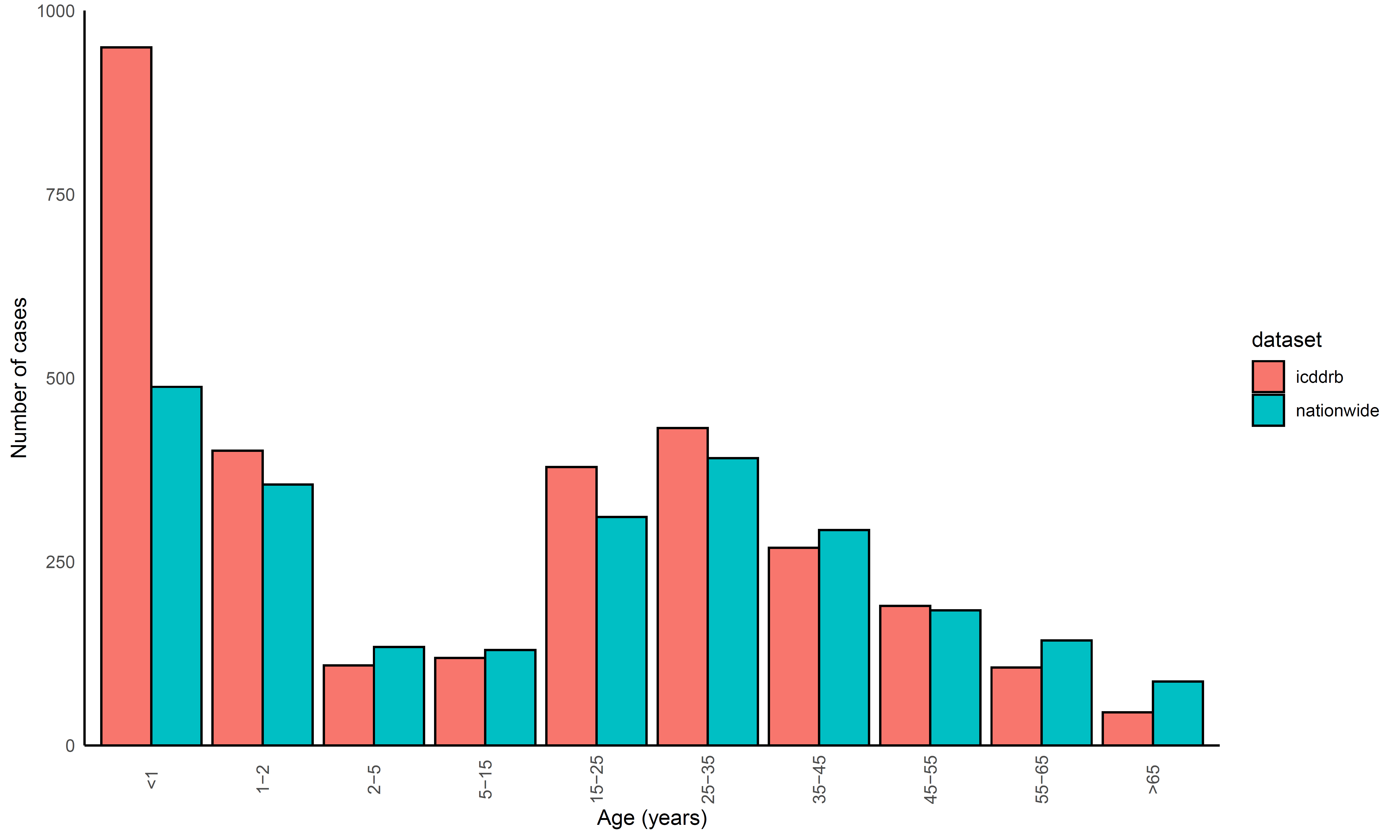


Supplemental Figure 2A. Prevalence of pathogens in diarrhea cases by surveillance network and age category. All pathogens detected in at least 1% of cases are shown.


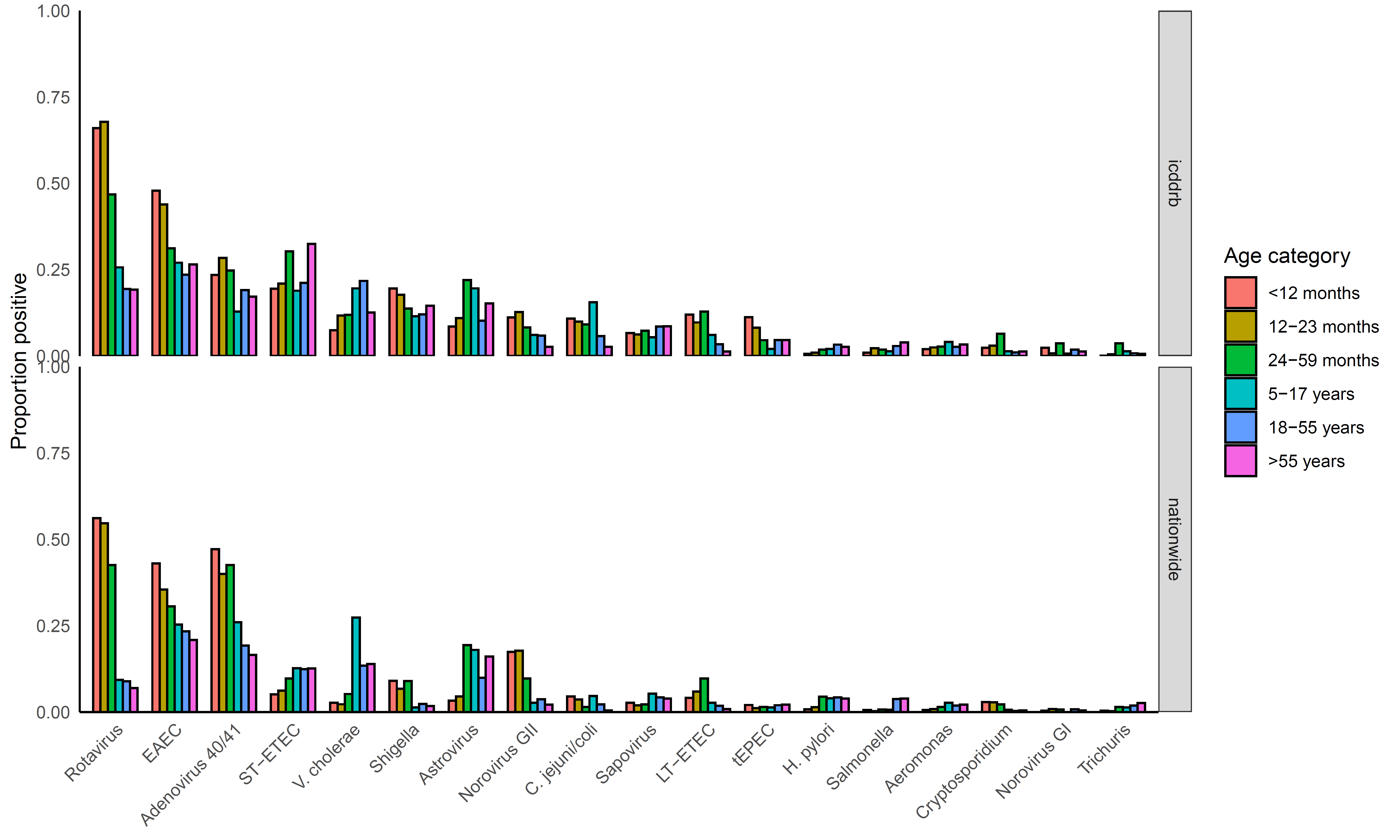


Supplemental Figure 2B. Prevalence of pathogens in non-diarrheal controls by surveillance network and age category. All pathogens detected in at least 1% of cases are shown.


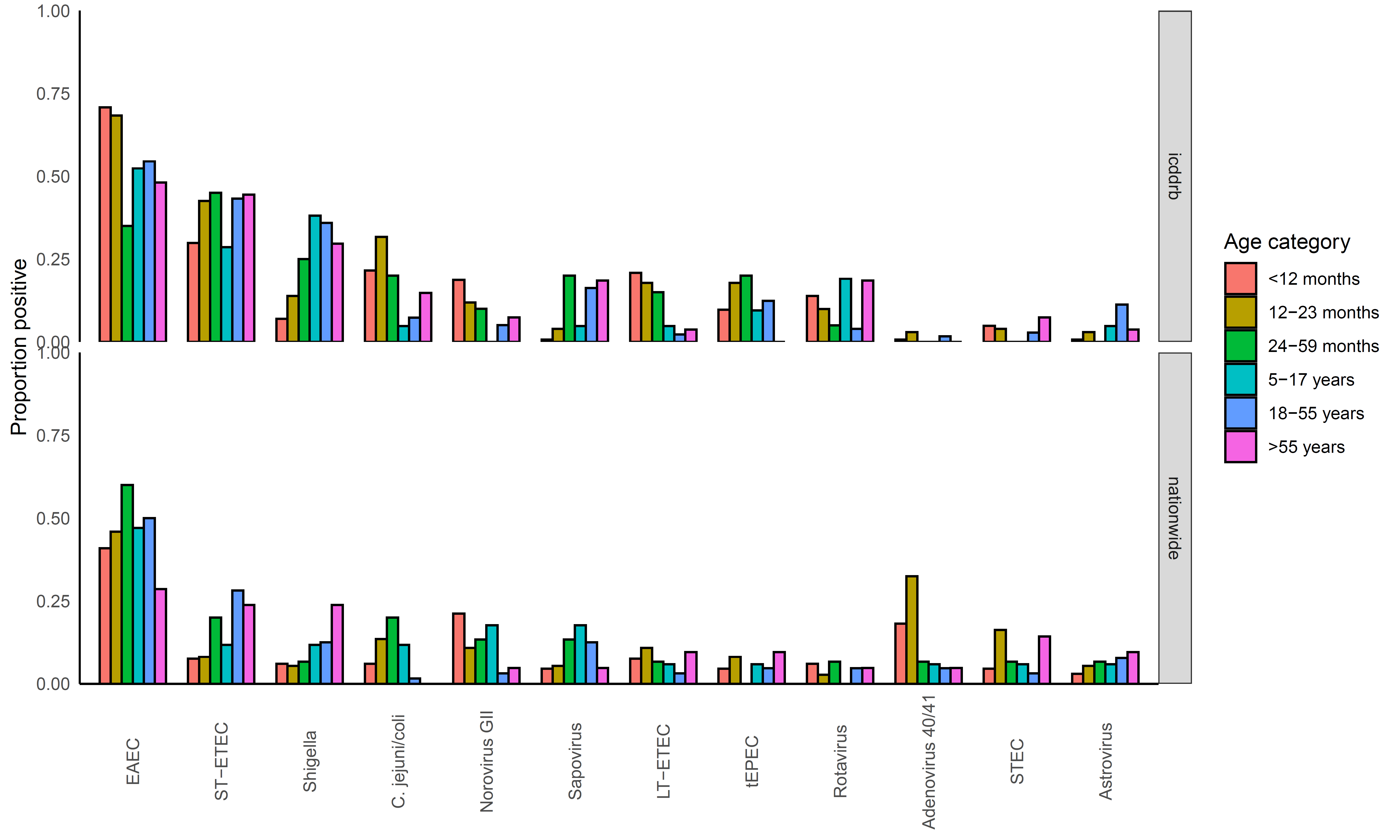


Supplemental Figure 3. Population attributable fractions by dehydration status.


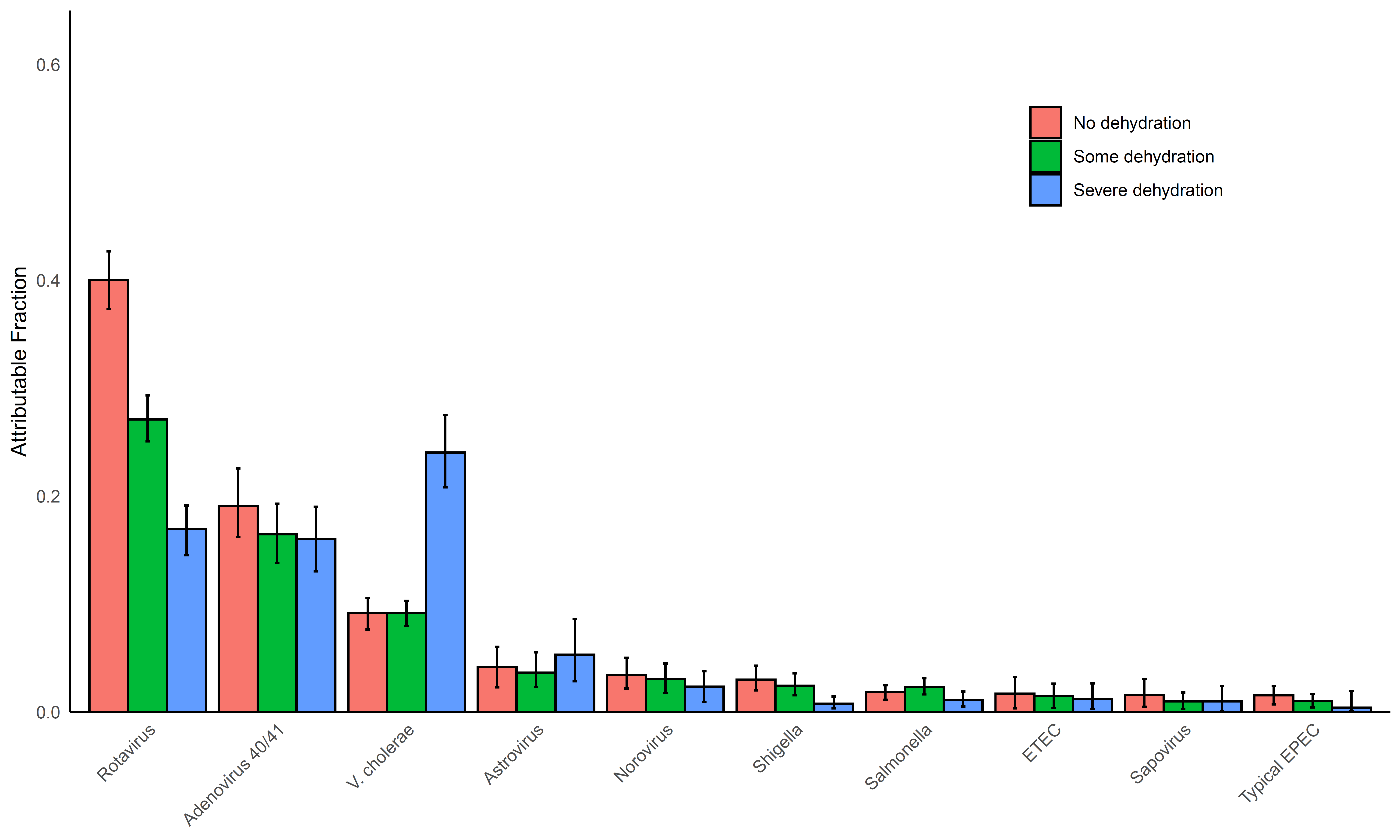


Supplemental Figure 4. Seasonal variation of etiology-specific diarrhea. The number of pathogen-attributable episodes for each calendar week was modeled using a Poisson model with a Fourier series.


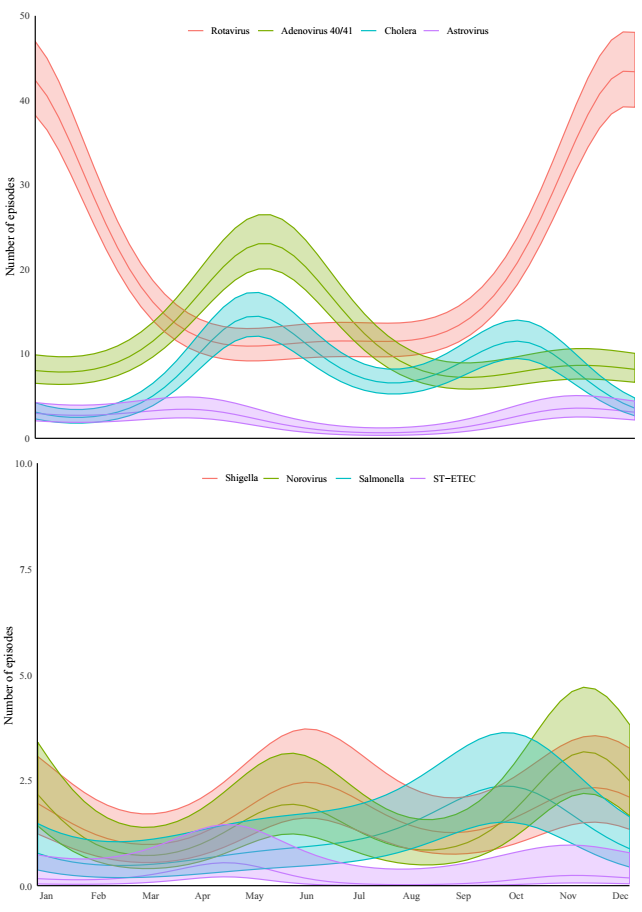


Supplemental Figure 5. Comparison of attributable fraction estimates using intrinsic (from this study) and extrinsic (from MAL-ED and GEMS studies) controls for children 0-59 months of age (n = 2437).


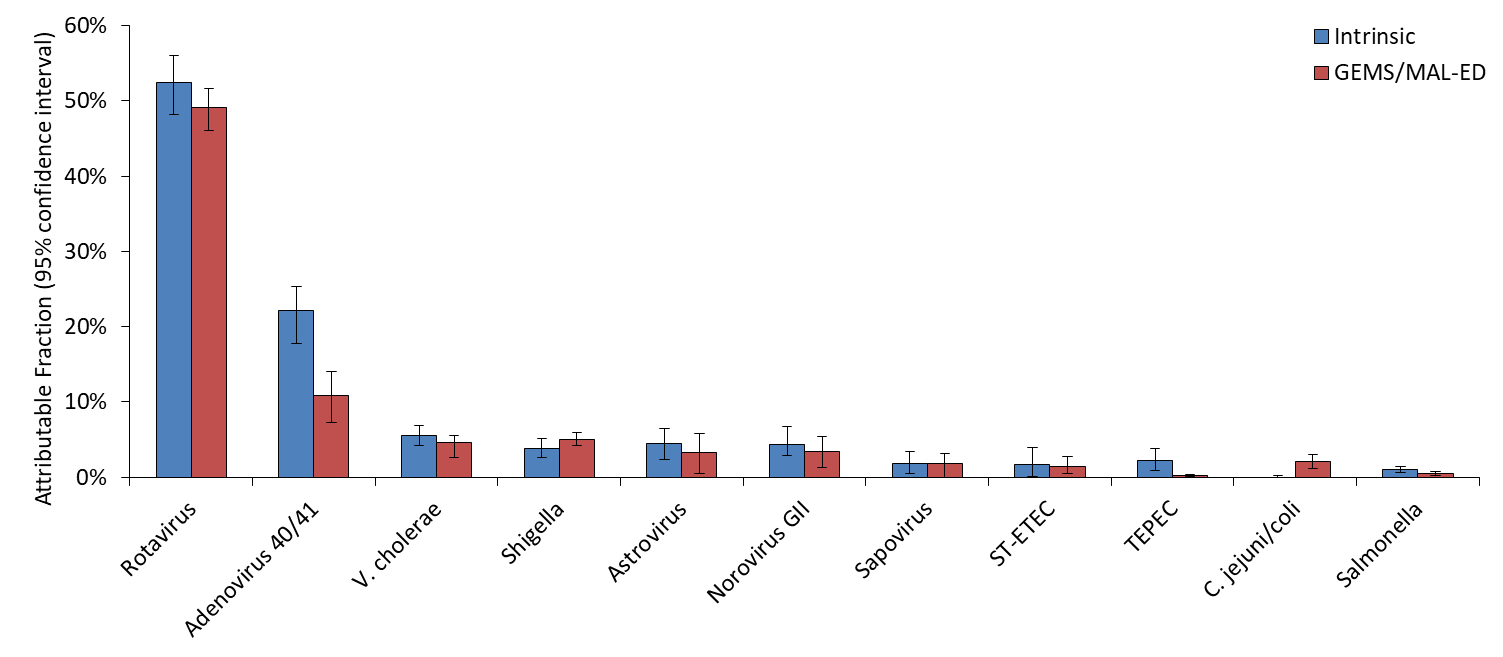

Supplement: ciaa840_suppl_Supplementary_Material [file ciaa840_suppl_supplementary_material.docx]
